# Supplementary material for: Effectiveness and Safety of Ayurvedic Medicines in Type 2 Diabetes Mellitus Management: A Systematic Review and Meta-Analysis
Source: Front Pharmacol. 2022 Jun 8;13:821810. doi: 10.3389/fphar.2022.821810 (PMC9213670; doi:10.3389/fphar.2022.821810)
Supplement: Supplementary file 1 [file DataSheet1.zip › Supplementary Material/Forest and Funnel Plots/Tinospora cordifolia/Tinospora cordifolia.pdf]

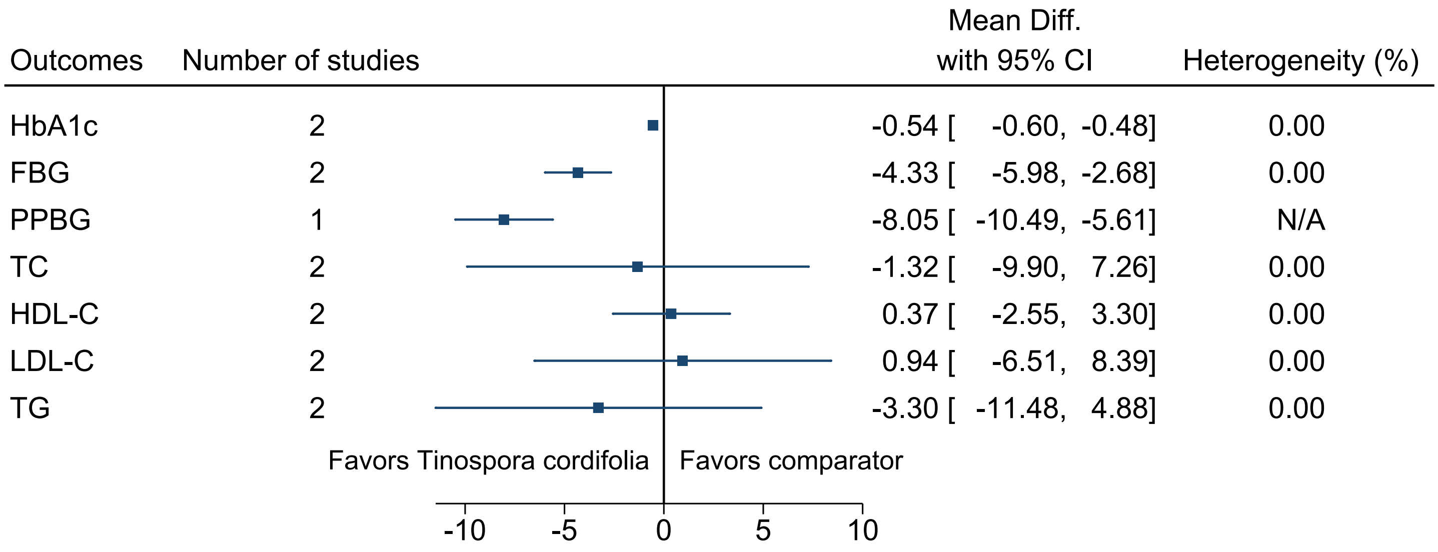

Outcomes
Number of studies
Mean Diff. with 95% CI
Heterogeneity (%)
HbA1c 2 -0.54 [-0.60, -0.48] 0.00
FBG 2 -4.33 [-5.98, -2.68] 0.00
PPBG 1 -8.05 [-10.49, -5.61] N/A
TC 2 -1.32 [-9.90, 7.26] 0.00
HDL-C 2 0.37 [-2.55, 3.30] 0.00
LDL-C 2 0.94 [-6.51, 8.39] 0.00
TG 2 -3.30 [-11.48, 4.88] 0.00
Favors Tinospora cordifolia
Favors comparator
-10
-5
0
5
10
